# Overexpression of CXCR7 accelerates tumor growth and metastasis of lung cancer cells

**DOI:** 10.1186/s12931-020-01518-6

**Published:** 2020-10-31

**Authors:** Huan Liu, Qian Cheng, Dong-sheng Xu, Wen Wang, Zheng Fang, Dong-dong Xue, Ya Zheng, Alex H. Chang, Yan-jun Lei

**Affiliations:** 1 Department of Traditional Chinese Medicine, Xijing Hospital Affiliated to the Fourth Military Medical University, Xi’an, 710032 China; 2 Department of Immunology and Microbiology, School of Basic Medical Science, Xi’an Jiaotong University Health Science Center, Xi’an, 710061 China; 3 Department of Anesthesiology, Cancer Hospital Affiliated to Fudan University, Shanghai, 200032 China; 4 Institute of Rehabilitation Medicine, Shanghai University of Traditional Chinese Medicine, Shanghai, China; 5 Department of Anesthesiology, Huadong Hospital Affiliated to Fudan University, Shanghai, 200040 China; 6 Department of Hepatobiliary Surgery, Hebei General Hospital, Shijiazhuang, 050051 China; 7 Rehabilitation Section, Spine Surgery Division of Department of Orthopedics, Tongji Hospital Affiliated to Tongji University School of Medicine, Shanghai, 200065 China; 8 Clinical Translational Research Center, Shanghai Pulmonary Hospital, Tongji University School of Medicine, Shanghai, 200438 China

**Keywords:** CXCL12/SDF-1, CXCR4, CXCR7, Overexpression, Invasion, Metastasis, Lung cancer

## Abstract

**Background:**

Under physiological conditions, CXCL12 modulates cell proliferation, survival, angiogenesis, and migration mainly through CXCR4. Interestingly, the newly discovered receptor CXCR7 for CXCL12 is highly expressed in many tumor cells as well as tumor-associated blood vessels, although the level of CXCR7 in normal cells is low. Recently, many studies have suggested that CXCR7 promotes cell growth and metastasis in more than 20 human malignancies, among which lung cancer is the leading cause of cancer-associated deaths worldwide. Thus, the mechanism of CXCR7 in the progression of lung cancer is urgently needed.

**Methods:**

First, we explored CXCR4 and CXCR7 expression in human lung cancer specimens and cell lines by immunohistochemistry, western blot and flow cytometry. Then, we chose the human lung adenocarcinoma cell line A549 that stably overexpressed CXCR7 through the way of lentivirus-mediated transduction. Next, “wound healing” assay and transwell assay were applied to compare the cell migration and invasion ability, and stripe assay was used to evaluate the cell polarization. Last, our team established a mouse xenograft model of human lung cancer and monitored tumor proliferation and metastasis by firefly luciferase bioluminescence imaging in SCID/Beige mice.

**Results:**

In clinical lung cancer samples, CXCR7 expression was almost not detected in normal tissue but upregulated in lung tumor tissue, whereas, CXCR4 was highly expressed in both normal and tumor tissues. Furthermore, overexpression of CXCR7 enhanced A549 cell migration and polarization in vitro. Besides, mouse xenograft model of human lung cancer showed that CXCR7 promoted primary lung tumor’s growth and metastasis to the second organ, such as liver or bone marrow in SCID/Beige mice in vivo.

**Conclusions:**

This study describes the multiple functions of CXCR7 in lung cancer. Thus, these results suggest that CXCR7 may be a malignancy marker and may provide a novel target for anticancer therapy.

## Background

The incidence of lung cancer ranks the top place in all kinds of malignant tumors worldwide and is progressively increasing year by year, with adenocarcinoma accounting for the most prevalent histological type. Lung cancer is also the most leading cause of death in men and the second cause of cancer-associated death in women worldwide [1]. Metastasis in lung cancer is the major reason leading to mortality of lung cancer patients. Although the application of the Lung Screening Trial (low dose helical computed tomography, LDCT) with chest radiography allows lung cancer to be diagnosed at an early stage, the prognosis of metastatic lung cancer is still unpromising even if combining surgery with radiotherapy, chemotherapy, immunotherapy and gene-targeted drug therapy [2, 3].

Chemokines are a superfamily of chemoattractant cytokines with diversity of biological and pathological functions, relating to immunocyte migration, hematopoietic stem cells homing, angiogenesis and tumor progression. So far, over 50 chemokines have been characterized, and they are divided into 4 classes (CXC, CX3C, CC, and C) based on the position of 4 conserved cysteine residues [4]. Chemokine receptors are seven-span transmembrane receptors coupled with G-proteins that are major regulators of cellular trafficking. Binding of chemokines to their receptors initiates a cascade of many cellular downstream signaling transduction pathways, including cyclic adenosine monophosphate-protein kinase A (cAMP-PKA), phosphatidylinositol and calcium fluxes mobilization or protein kinase C (PI-Ca^2+^/PKC) and cyclic guanosine monophosphate-protein kinase G (cGMP-PKG) signaling pathway [5].

The chemokine CXCL12, also known as stromal cell-derived factor-1(SDF-1), has been identified as playing a crucial role in cell migration, angiogenesis, tumor cells proliferation and metastasis, as well as in autoimmune diseases such as rheumatoid arthritis (RA) [6, 7]. It was first cloned from a bone marrow-derived stromal cell line and was later identified as a pre-B-cell growth stimulating factor which matured to be antibody-secreting cell. CXCL12 is widely expressed in a range of tissue types and mainly secreted by stromal and endothelial cells. Elevation of CXCL12 expression is followed by tissue damages such as hypoxia, ischemia, reperfusion injury, irradiation and chemotherapy related damages, which may act as chemoattractant of tissue-committed stem cells (TCSCs) participating in tissue repair [5]. The receptor for the CXCL12 is the C-X-C chemokine receptor type 4 (CXCR4), a typical seven transmembrane G-protein coupled receptor (GPCR). CXCR4 has received extensive attention because it serves as a co-receptor for entry of T-tropic human immunodeficiency viruses (HIV) into CD4^+^ T cells [8]. During development, many researches have shown that CXCR4 is expressed in a broad variety of tissues, including the immune, circulatory and central nervous systems, functioning in multiple biological processes. For instance, in the immune system, CXCR4 involves in the differentiation and development of leukocytes in peripheral blood and hematopoietic progenitor cells in bone marrow and facilitates immune cells to function like migration and adhesion to endothelial cells [9]. Furthermore, many studies have determined that CXCL12/CXCR4 axis potentiates proliferation, angiogenesis, invasion and migration of various cancers, including glioma, colorectal carcinoma, renal cancer, pancreatic cancer and ovarian cancer [10–14].

In addition to CXCR4, CXCR7 has been identified as a new receptor for CXCL12. Being originally classified as a 7-transmembrane orphan receptor, CXCR7 was identified to be a CXCL12 receptor with a ten-fold higher binding affinity toward CXCL12 than CXCR4 [15, 16]. In contrast to CXCR4, CXCR7 can’t activate G protein kinase due to the mutation of the “DRAILIV” motif, and thus is unable to trigger G-protein-dependent signaling [17]. Actually, when CXCR7 is activated by CXCL12, the β-arrestin pathway is activated and CXCL12 scavenging is observed. Besides, CXCR4 and CXCR7 can also form heterodimers, whereby CXCR7 regulates the signaling of CXCR4/G-protein complexes depending on different physiological conditions [4]. However, with development, literature findings have highlighted that CXCR7 participates in the transduction of some signaling pathways without relying on CXCR4, including PLC / MAPK, ERK1 / 2, STAT3, and AKT pathways. These signaling pathways unveil a decisive role in the proliferation, adhesion, invasion and metastasis of tumor cells [18–20]. It is worth noting that CXCR7 is highly expressed in many tumor cells and tumor-associated blood vessels, whereas it is undetected in blood vessels associated with normal tissues [21, 22]. High expression CXCL12 in metastatic lymph nodes (MLN) is observed and confirmed as independent prognostic factor in lymph-node-positive non-small-cell lung cancer (NSCLC) patients [23]. Also, in p-stage I NSCLC patients, high CXCR7-expressing patients have a poorer 5-year disease-free survival rate (DFS) and higher postoperative metastatic recurrence (Rec-Distant) than low CXCR7-expressing patients [24]. However, the specific role of CXCR7 in the proliferation, migration, invasion and metastasis of lung cancer cells has not been systematically reported.

Mounting evidences supported that the CXCL12/ CXCR4 / CXCR7 axis was related to multiple types of solid tumors and tumor cells. CXCR7 expression was controlled by the particular microenvironment of tumors [25]. In this study, we are the first to attempt to systematically investigate the role of CXCR7 independent of CXCR4 in migration, invasion and polarization of lung cancer cell line A549 in vitro and tumor growth and metastasis by establishing animal lung cancer model in vivo.

## Materials and methods

### Clinical tissue samples

In this study, clinical tissue samples were surgical resection specimens obtained from Huadong Hospital Affiliated to Fudan University. Written informed consent was obtained from all patients participating in this study. Clinical diagnosis of all patients were confirmed by pathological examination, laboratory tests and clinical manifestation according to the international association for the study of lung cancer (IASLC) diagnosis criteria.

### Cell lines and culture

The human lung adenocarcinoma cells A549 and Large-cell lung cancer cell line H460 (ATCC; Manassas, VA, USA) were purchased from Shanghai cell bank of Chinese academy of sciences. These cell lines were cultured in Dulbecco’s modified Eagle’s medium (Thermo Scientific Hyclone, South Logan, UT, USA) supplemented with 10% fetal bovine serum (FBS) (Gibco, Life Technologies, Carlsbad, CA, USA) and 1% penicillin/streptomycin (Hyclone). Human umbilical vein endothelial cells (HUVECs) and its completed medium were obtained from AllCells biological technology (Shanghai) co., LTD. And all these cell lines were maintained in a 37 °C incubator with 5% CO_2_.

### Lentivirus-mediated overexpression of CXCR7 and transduction

psPAX2 (gag-pol expressor) and pMD2G (VSV-G expressor) were used as the lentiviral packing vector, and pRRL.EF1α plasmid with human CXCR7(NC_000002.12) were synthesized by Genscript biological technology (Nanjing, China) co., LTD. Lentiviruses were produced by co-transfection of HEK293T cells with psPAX2, pMD2G and pRRL.EF1α-hCXCR7 vectors. For overexpression, A549 cells were transduced with the collected lentiviruses at 1:5 and 1:20 two different concentrations in the presence of 8μg/ml Polybrene (Sigma-Aldrich, St Louis, MO, USA). In order to further enrich CXCR7 expressing A549 cells, lentivirus transducted cells were digested with 0.25% EDTA-Trypsin (Sigma-Aldrich) and washed with FACS buffer (PBS + 2%FBS) for two times. Subsequently, cells were then stained with 2 μl PE-conjugated antiCXCR7 antibodies (Clone#11G8, R&D Systems, Minneapolis, MN) on ice for 30 min and washed with FACS buffer for two times. Finally, cells were resuspended in serum-free DMEM medium and filtered through a filter of 40 μm pore size (BD Biosciences). All procedures were underwent in a sterile environment. FACS sorting was performed (FACS Aria, BD Biosciences, San Jose, CA) and analyzed on a FACS machine (Calibur, BD, Franklin Lakes, NJ).

### Wound healing assay

Cells were seeded in ibidi culture-insert (7000 cells per well) in 70 μl DMEM (serum free) with CXCL12 (100 ng/ml, R&D Systems) in the presence or absence of CXCR7 inhibitor CCX771(0.5 μM) (Chemo Centryx, Mountain View, CA), and pre-incubated overnight in 37 °C 5%CO_2_, and then gently removed the culture-insert by using sterile tweezers, filled the used wells with free medium, and photograph it in 0,12 as well as 24 h. and compared the width in different group and timepoint.

### Transwell assay

Transwell assay inserts (24 mm diameter, 8 μm pores, Costar, Corning, NY, USA) were placed in 24-well plates. Cells (2 × 10^4^) were resuspended in 1% FBS-DMEM medium and placed in the upper chamber, whereas 20% FBS-DMEM alone or containing 100 ng/ml CXCL12 (R&D Systems) or 0.5 μM CCX771 (Chemo Centryx) was added to the lower chamber. After incubation for 12 h, the inserts were fixed by methanol and DAPI. Non-invading cells were mechanically wiped using cotton swabs. The invading cells were counted with Zeiss 710 confocal microscope (× 100 magnification).

### Polarization

Polydimethylsiloxane (PDMS) was sealed on PDL-coated glass bottom dishes (35 mm dish with 20 mm bottom well), and microchannels were formed between the PDMS mold and well. The mixture of BSA and CXCL12 or BSA alone was added to one end of the microchannels, and vacuum was applied to the other end of the microchannels to ensure BSA/CXCL12 or BSA filled in all the microchannels. PDMS molds were removed after drying overnight. Cells were then fixed using 4% paraformaldehyde (PFA), and permeabilized with 0.4% Triton-X in PBS on dishes. Cells were incubated with primary antibodies overnight, and Cultures were washed and then incubated with phalloidin and corresponding secondary antibodies for 1 h at room temperature. Nuclear DNA was labeled with DAPI for 2 min after the secondary antibody at room temperature. Cultures were washed with PBS thrice then images were taken by a Zeiss confocal microscope.

### Western blot

Certain number of cells were collected and total cellular proteins were extracted in M-per(R) Mammalian Protein Extraction Reagent (Thermo Scientific). Cellular proteins were separated by 10% sodium dodecyl sulfate-polyacrylamide gel electrophoresis (SDS-PAGE) electrophoresis and transferred to poly-vinylidene fluoride membranes (Millipore; Bedford, MA) for immunoblotting. After blocking with 5% skim milk (BD Biosciences), the membrane was probed with the primary antibodies rabbit monoclonal to GPCR RDC1 (abcam, Cambridge, MA.ab138509,1:1000 diluted) overnight at 4 °C. After washing, membranes were labeled with the horseradish peroxidase conjugated goat anti-rabbit secondary antibodies (R&D Systems,1:4000) for 1 h at room temperature. The immunoblots were colored by using an ECL Kit (Millipore). Anti-actin was used for loading control (Sigma-Aldrich). Blots were detected and imaged by Image Lab (Bio-Rad).

### Flow cytometry

For cell surface chemokine receptor detection, approximate 1 × 10^5^ cells were harvested,washed and suspended in FACS buffer (PBS + 2%FBS) in 50 μl volume. Cells were then stained with 2 μl Phycoerythrin (PE)-conjugated anti-CXCR7 antibodies (R&D Systems) or APC-conjugated anti-CXCR4 antibody (Clone#12G5, BD Systems) for 30 min in a dark place on ice, and mouse anti-human IgG1 served as isotype control. Stainned cells were washed twice with FACS buffer, resuspended in FACS buffer in a proper volume (250 ~ 350 μl) for analysis. Ten thousands cells from each sample were evaluated for fluorescence detection using FACS machine (Calibur, BD, Franklin Lakes, NJ), and the data were analyzed with FlowJo 7.6 software.

### Xenograft model and bioluminescence imaging

The SCID/Beige (Severe Combined Immune-deficiency/Beige, 6–8 weeks of age, female) mice were purchased from SLRC Laboratory Animal (Shanghai, China) co., LTD. A total of 1.8 × 10^6^ A549-GFPLuc or A549-GFPLuc-CXCR7 lung adenocarcinoma cells were injected intravenously via a tail vein in 200 μl of serum-free DMEM medium. Bioluminescence imaging was used to monitor cell proliferation and metastasis every week. For bioluminescence imaging, mice were injected intraperitoneally with D-luciferin at a dose of 150 mg/kg. After injection of D-luciferin, the mice were anesthetized with isoflurane/oxygen and placed on the imaging platform. The bioluminescence signals were monitored using an IVIS-200 bio-photonic imager (LB983, Bertold Technologies) within 20 min. The data were analyzed using the “IndiGO” software and total photon flux emission in the regions of interest were quantified.

### Immunohistochemistry and immunofluorescence

Lung tumor samples were dissected from mice, fixed in 4% paraformaldehyde, and embedded in paraffin for immunohistochemistry, and sectioned at 4 μm intervals. Standard IHC techniques were performed according to the manufacturer’s recommendations. The slides were deparaffinized in xylene and rehydrated through a series of dilutions of alcohol. The slides were then placed in EDTA epitope retrieval buffer (pH 8.0) in a microwave for two 8 min sessions. After blocking with 3% H_2_O_2_ and 3% BSA, the treated slides were then incubated with the primary antibodies against CXCR7 (gift of ChemoCentryx,1:200) for overnight at 4 °C and horseradish peroxidase conjugated goat anti-mouse secondary antibodies (Dako North America Inc., Carpinteria, CA, USA) for 1 h at room temperature. DAB Color Development Kit were used for detection of the bound antibodies. Furthermore, Nuclei were counterstained with hematoxylin, washed, dehydrated and mounted. The stained cells were observed by using a bright-field microscope in each experimental group, imaged and analyzed. Nuclei were stained in blue and the CXCR7 expression positive cells in brown yellow. For immunofluorescence analysis, lung samples were embedded in OCT reagent (Sakura) and sectioned at 10 μm intervals. The frozen sections were fixed with cold acetone for 10 min and rinsed with PBS (PH7.4) for three 5 min sessions. Finally, nuclei were counterstained using DAPI. Slides were mounted using resistance of fluorescence quenching reagent and then observed by inverted fluorescence microscope (Nikon Eclipse Ti-SR).

### Statistical analysis

Data were shown as mean values with SEM. Statistically significant differences were analyzed by Graphpad Prism software (Avenida de la Playa, La Jolla, CA, USA). Wound healing assay and Transwell assay experiments were performed 3 times each, and representative data were presented and determined by cell migration distance. Photon flux values of each group were compared by using the t-test. For all graphs, *P < 0.05; **P < 0.01; ***P < 0.001.

## Results

### CXCR7 expression was upregulated in lung tumor tissue compared with normal tissue, whereas CXCR4 expression was not significantly changed

**Fig. 1:**
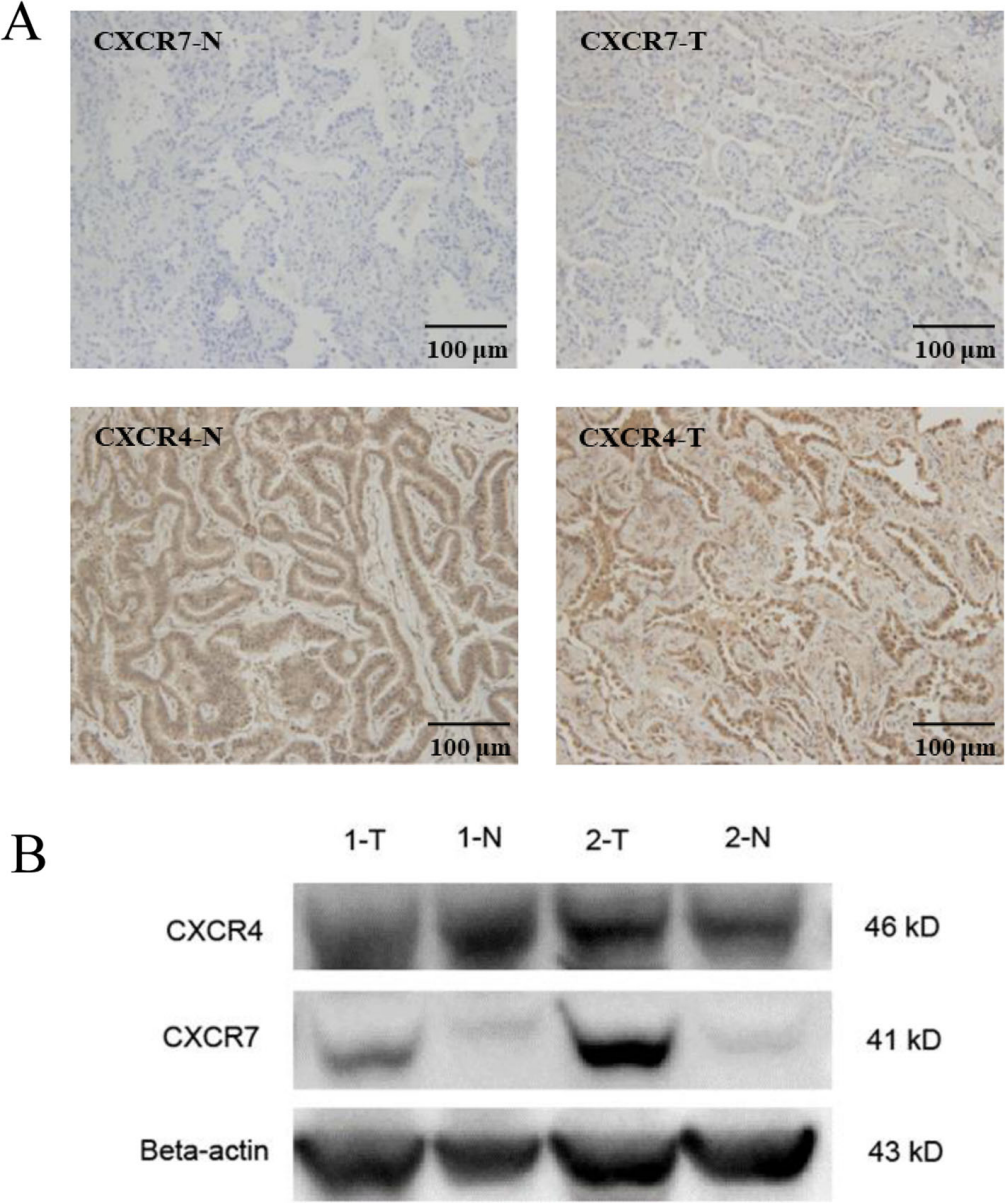
CXCR7 and CXCR4 expression in lung tumor tissue. **a.** Lung tumor samples were subjected to immunohistochemical staining for CXCR7(upper) and CXCR4(lower) expression, para-tumor tissue as normal control (*n* = 4) (Magnification: 200×). **b.** Lung tumor tissue and para-tumor tissue were lysed and analyzed for CXCR7 and CXCR4 expression by western blot. β-actin was used as a loading control (*n* = 2)

**Fig. 2:**
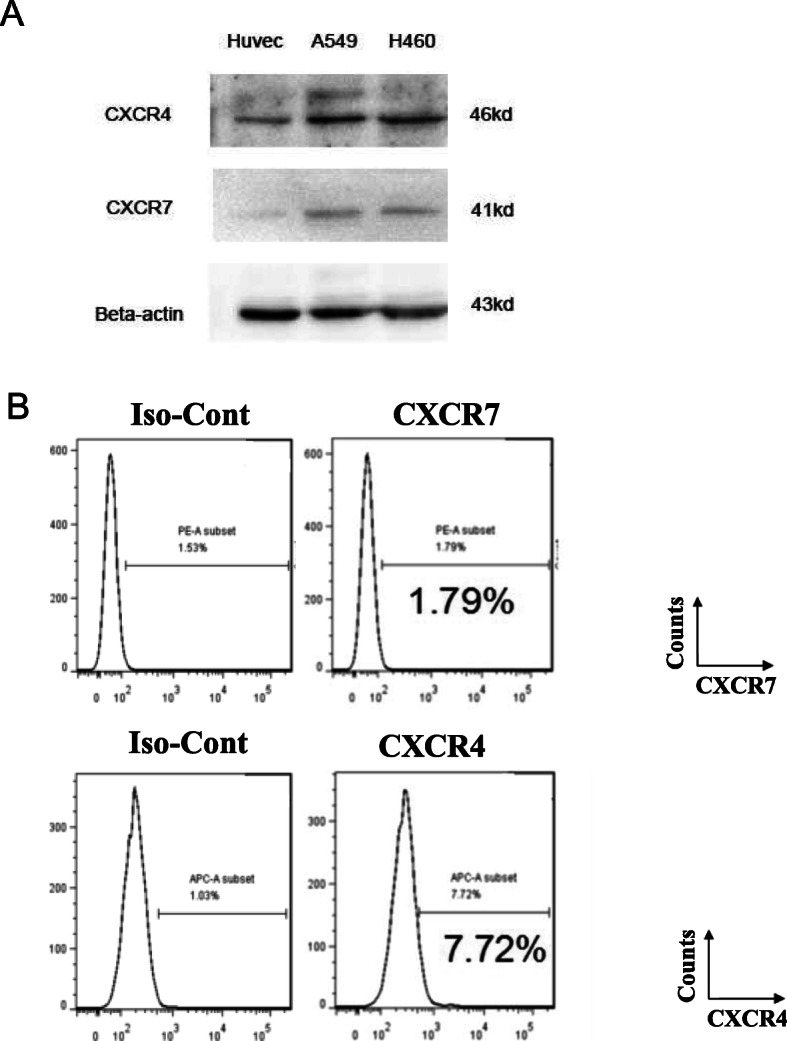
CXCR7 and CXCR4 expression in lung cancer cell lines. **a.** Western blot was performed for CXCR7 and CXCR4 protein expression in established human lung cancer cell lines. Human umbilical vein endothelial cells (HUVEC) were used as a normal control. β-actin was used as a loading control. **b.** FACS analysis of CXCR7 and CXCR4 protein expression on the cell membrane of A549 lung cancer cell line. CXCR7 protein expression was evaluated by FACS analysis using PE-conjugated anti-CXCR7 antibody. CXCR4 protein expression using APC-conjugated anti-CXCR4 antibody

The clinical lung adenocarcinoma tissue samples and para-tumor tissue samples gained from Huadong Hospital Affiliated to Fudan University, Shanghai, China. To compare CXCR7 expression level, we used lung adenocarcinoma tissue sample as experimental group while the para-tumor tissue sample as control group. Immunohistochemical results showed that the expression of CXCR7 was barely observed in normal tissue, but upregulated in lung cancer tissue (Fig. 1a). CXCR4 expression was highly expressed in both normal and lung cancer tissue, and seemed to be higher in cancer tissue (Fig. 1a). It is worth mentioning that CXCX7 and CXCR4 staining were found mainly in pulmonary epithelial cells, but less in the lung stromal cells. Meanwhile, the results of the expression of CXCR7 and CXCR4 in normal tissue and lung cancer tissue by western blot was consistent with that of immunohistochemical staining (Fig. 1b). Furthermore, western blot was performed for CXCR7 and CXCR4 protein expression in human lung adenocarcinoma cell line A549 and large cell lung cancer cell line H460.Human umbilical vein endothelial cells (HUVEC) were used as a normal control. Similarly, CXCR7 was scarcely expressed in HUVEC, but up-regulated in A549 and H460, while CXCR4 was expressed in HUVEC, A549 and H460 but was higher in lung cancer cell lines (Fig. 2a). Next, human lung adenocarcinoma cell line A549 were selected for subsequent in vitro and in vivo studies. Flow cytometry was used to detect the expression levels of CXCR7 and CXCR4 on the surface of A549 cells, and the results showed that there was almost no expression of CXCR7 and CXCR4 on the surface of A549 cells (Fig. 2b). It has been reported that the chemokine receptor on the cell surface can’t be detected because its ligand occupies the binding site of the flow antibody and the chemokine receptor, which can be detected after the ligand receptor is unbound by acid washing [5]. As to the expression of chemokine receptors, unfortunately, the expression of CXCR7 and CXCR4 could not be detected by flow cytometry after acid washing and permeabilization pretreatment of A549 cells (data not shown).

### Construction of A549 cells overexpressing CXCR7 (A549-CXCR7-O)

**Fig. 3:**
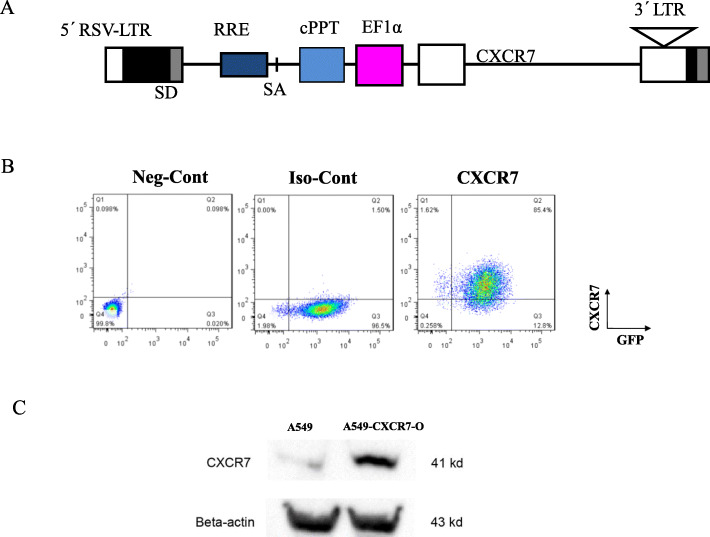
Overexpression of CXCR7 in A549 lung cancer cell line. **a.** The structure of CXCR7 overexpression vector. **b.** FACS analysis of CXCR7 protein expression in constructed A549-GFPLuc-CXCR7 overexpression lung cancer cell line. CXCR7 protein expression was evaluated by FACS analysis using PE-conjugated anti-CXCR7 antibody, and anti-IgG1 served as isotype control. Transduction of GFPLuc was used for report gene and firefly luciferase bioluminescence imaging of lung cancer animal model in vivo. **c.** Western blot was conducted to validate high-expressed CXCR7 in successful constructed A549-GFPLuc-CXCR7 overexpression lung cancer cell line. β-actin was used as a loading control

It is well known that chemokines usually exert physiological effects on the cell surface by binding to ligands. We tried to obtain a stable transfected A549 cell line (A549-CXCR7-O) overexpressing CXCR7 on the cell surface via lentiviral transfection by constructing an CXCR7-overexpressed vector--pRRL.EF1α-CXCR7 (Fig. 3a). A549 cells were infected with packaged lentivirus pRRL.EF1α-GFPLuc as control, or co-infected with pRRL.EF1α-GFPLuc plus pRRL.EF1α-CXCR7 to obtain CXCR7-overexpressing lung cancer cell line. Transduction of GFPLuc was used for report gene and firefly luciferase bioluminescence imaging of lung cancer animal model in vivo. In order to further enrich GFPLuc-CXCR7 expressing A549 cells, infected cells were then stained with PE-conjugated anti-CXCR7 antibody, and FACS sorting was performed to select double-positive (FITC and PE) population cells. Moreover, CXCR7 overexpression was confirmed by FACS (Fig. 3b) and western blot (Fig. 3c), indicating that forced CXCR7 overexpression is mainly expressed on the cell surface. Furthermore, in the purpose of comprehending cellular trafficking of CXCL12/CXCR7 axis, cultured A549-GFPLuc-CXCR7-O cells were stimulated with CXCL12 (100 ng/ml) in a time course. It was a pity that there was no clear trend that CXCR7 transferred from cell membrane to intracellular and then back to the cell membrane. It may be explained that this recycling machinery could only cope with endogenously expressed, but not with forcedly, overexpressed CXCR7, which could be supported by the research of other group [46] (data not shown).

### Forced CXCR7 overexpression enhanced A549 cell migration, invasion and polarization

**Fig. 4:**
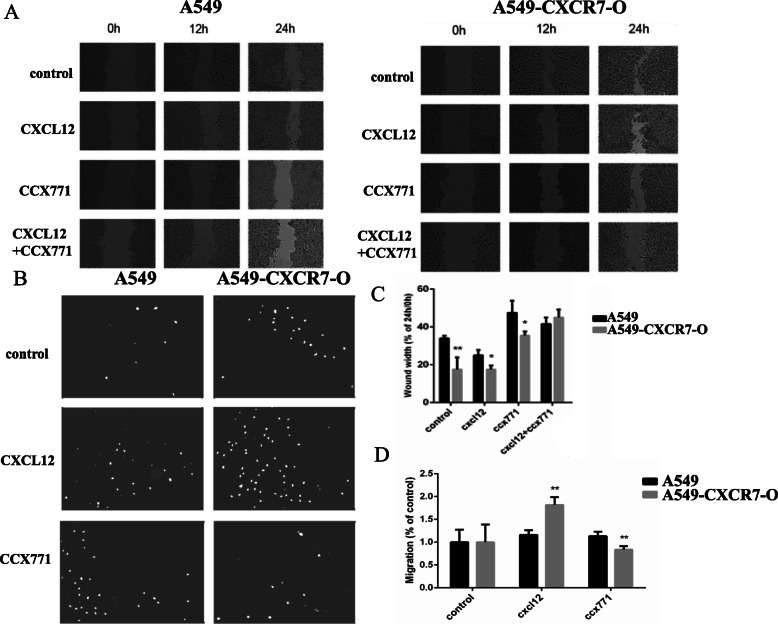
CXCL12 and CCX771 modulates CXCR7-dependent A549 cell migration and invasion. A549 and A549-CXCR7-O cells were incubated in 12 well plate with ibidi labware untill the confluence is 90%. Before removing the Culture–Insert, cells were serum starved for 12 h. Then a cell–free gap of approximate 500 μm is created after removing the Culture–Insert. And the closure of the wounds was monitored in the presence or absence of CXCL12 (100 ng/ml), CCX771(0.5 μM) or both by microscopy after 0, 12 and 24 h. **a.** Imaging and **c.** Quantitative analysis of percentage of wound closure.1 × 10^5^ of A549 or A549-CXCR7-O cells were seeded per well, and pre-incubated for 12 h, treated in different group as indicated for 12 h. **b.** Imaging and **d.** Quantitative analysis of number of migrated cells. *, *p*<0.05, **, *p*<0.01

Wound healing assay was used to evaluate the cell migration ability. A549 and A549-CXCR7-O cells were incubated in 12 well plate with ibidi labware untill the confluence is 90%. Before removing the Culture–Insert, cells were serum starved for 12 h. Then a cell–free gap of approximate 500 μm is created after removing the Culture–Insert. And the closure of the wounds was monitored in the presence or absence of CXCL12 (100 ng/ml), CCX771(0.5 μM) or both by microscopy after 0, 12 and 24 h (Fig. 4a). The results showed that at 24 h after insert removal, the “wound healing” percentage of A549-CXCR7-O cells was significantly higher than that of A549 cells (**, *p* < 0.01). Under the role of CXCL12 and CCX771 respectively, it can be seen that the percentage of “wound healing” of A549-CXCR7-O cells is still higher than that of A549 cells (*, *p* < 0.05). But at the present of both CXCL12 and CCX771, the difference in migration speed between the two groups of cells was not statistically significant (Fig. 4b).

We used transwell assay to observe the cell invasion ability. 1 × 10^5^ of A549 or A549-CXCR7-O cells were seeded per well, and pre-incubated for 12 h, treated in different group as indicated for 12 h (Fig. 4c). In the presence of CXCL12, the invasion ability of A549-CXCR7-O cells was significantly higher than that of A549 cells (**, *p* < 0.01), while after using CCX771, because of inhibiting the function of CXCR7 by CCX771, the invasion ability of A549-CXCR7-O cells was significantly reduced, which was lower than that of A549 cells (**, *p* < 0.01) (Fig. 4d).

**Fig. 5:**
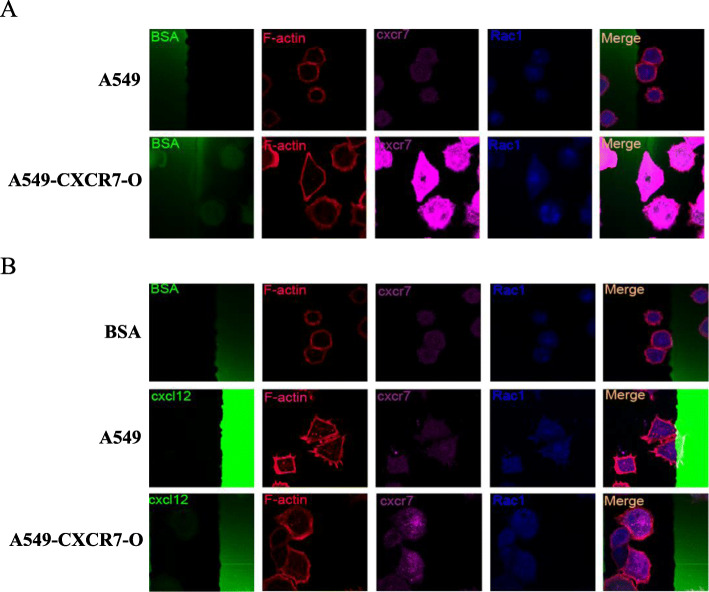
CXCR7 overexpression promotes A549 cell polarization. **a.** Morphology of A549 and A549-CXCR7-O cell lines in stripe assay. **b.** Polarization of A549 and A549-CXCR7-O cell lines in stripe assay

A549 cells were epithelial-like human lung adenocarcinoma cells. After A549 cells overexpressed CXCR7, some cells showed spindle deformation. At the same time, cells were stained with Rac1 protein, which was involved in cell migration, cytoskeleton regulation, and cell cycle regulation (Fig. 5a). Moreover, under CXCL12 stimulation, more cells could be observed to undergo spindle deformation, and even spine-like small protrusions were formed on the cell surface. Compared with A549 cells, A549-CXCR7-O cells appeared to be larger in volume and cell polarization was more pronounced (Fig. 5b).

### CXCR7 overexpression in A549 cells not only promoted primary lung tumor growth but also metastasis

**Fig. 6:**
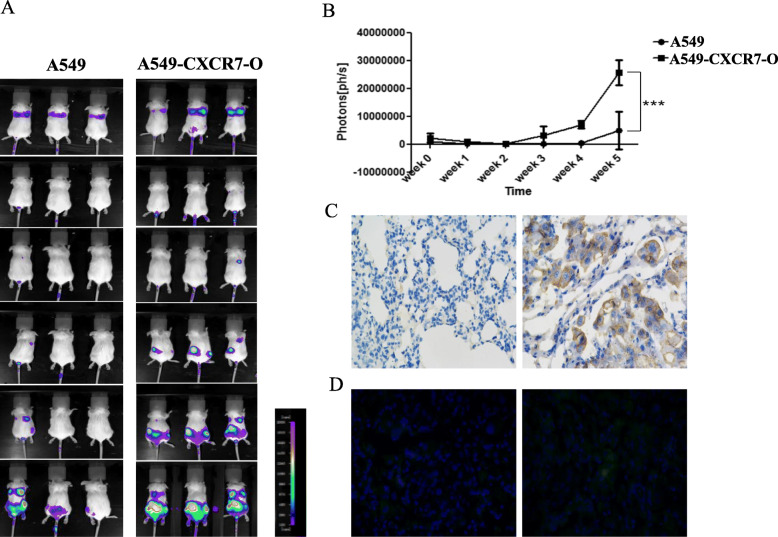
Overexpression of CXCR7 promotes growth of lung cancer cells and metastases in SCID/Beige mice. Mice were injected intravenously with lung cancer cells of A549-GFPL or A549-GFPL -CXCR7-O cells via tail vein injection. **a.** Continuous images are presented from firefly luciferase bioluminescence imaging for tumors every week until 5 weeks (From top to bottom). Scale bar depicts range of photon flux values as pseudocolor display with red and blue representing high and low values, respectively. **b.** Quantified data for photon flux from each group of mice. Graph displays mean values + SEM. ***, *p* < 0.001. **c.** Lung tissues from A549-GFPL (left) and A549-GFPL-CXCR7-O (right) cell-derived tumors were subjected to immunohistochemical staining for CXCR7.Nuclei were counterstained with hematoxylin (blue) (Magnification:200×). **d.** GFP fluorescence image. DAPI was used for nuclear detection (Magnification:200×)

For ensuring the credibility of experimental data in animal model, we first used limiting dilution method to obtain single colony from both A549-GFPLuc and A549-GFPLuc-CXCR7-O constructed lung cancer cell lines. Then we compared the bioluminescent intensity at the same conditions in vitro by microplate reader with each cultured single colony. Finally, single colony from both A549-GFPLuc and A549-GFPLuc-CXCR7-O cell lines having approximately bioluminescent intensity were selected and served as the subsequent animal experiments (data not shown). To establish live monitoring lung cancer mouse model, we injected A549-GFPLuc or A549-GFPLuc-CXCR7-O cells iv. via tail vein into SCID/Beige mice. Continuous images are conducted from firefly luciferase bioluminescence imaging for tumors with A549-GFPLuc or A549-GFPLuc-CXCR7-O cells every week until 5 weeks to investigate the extent to which CXCR7 affects growth and metastasis of cell-derived lung cancers. About 7 to 10 days after cell injection, there was an immune elimination process in mice body, which meant that human-originated cells were regarded as a “alien” and were cleared, leaving severe tumorigenicity population cells to grow up (Fig. 6a). The overall photon flux from each group of mice were calculated and exhibited significant difference between CXCR7-overexpressed group and control group(*P* < 0.001) (Fig. 6b). The outcome of live monitor imaging indicated that SCID/Beige mice injected with A549-GFPLuc-CXCR7-O cells formed larger primary tumors in lung position than control group at 28th day. Remarkably, SCID/Beige mice injected with A549-GFPLuc-CXCR7-O cells had an easier metastasis tendency from lung primary organ to other organs like liver and bone marrow. To demonstrate that lung cancer cells remaining relative distinguishing differences were due to the level of CXCR7 expression, lungs were removed from experimental mice and immunostained for CXCR7 expression, using the CXCR7 specific antibody 11G8 at 35th day after the last imaging. Importantly, CXCR7 expression was undetectable or at very low levels in lung tissues from A549-GFPLuc cell-derived tumor mice. Whereas, robust expression of CXCR7 was clearly detected in A549-GFPLuc-CXCR7-O cell-derived tumor lung tissue (Fig. 6c). In addition, GFP as a fluorescence marker could indirectly indicate tumor development. Thus, immunofluorescence was performed to trace GFP fluorescence. Coincident with the date from IHC, the fluorescence intensity in A549-GFPLuc cell-derived tumor lung tissue was weaken than in A549-GFPLuc-CXCR7-O and representative images were presented (Fig. 6d).

## Discussion

The interplay of chemokines and chemokine receptors in the tumor microenvironment is essential for the acquisition of the metastatic properties of cancer cells [26]. Previous work had focused on the role of CXCL12 and its receptor CXCR4 in tumor development and how they affected overall prognosis of cancer patients. Even CXCR4 antagonists had been carried out with AMD3100 (Plerixafor) as a prototypic CXCR4 antagonist which happened to be discovered, and had been approved by the US Food and Drug Administration (USFDA) for the treatment of lymphoma and multiple myeloma. Although CXCR4 plays a significant role in tumor growth, angiogenesis, invasion and metastasis, long-term blockade of CXCR4 might yield severe adverse effects relating to prenatal death in mice lacking CXCL12, as it may spare the normal tissues. There is a significant amount of accumulating evidence indicating that the alternate CXCL12 receptor, CXCR7, is highly expressed in the process of embryonic development, but it has been decreased after birth and low expressed in normal blood cells [27]. Most importantly, CXCR7 is mainly present in cancer cells and rarely in developed normal cells in adults. This is consistent with our results of CXCR7 and CXCR4 expression detected in lung cancer clinical samples and cell lines, as depicted in Figs. 1 and 2. Unlike CXCR4, CXCR7 is a non-classical GPCR that is unable to activate G proteins. The function of CXCR7 is considered to be mediated by: (a) recruiting β-arrestin-2; (b) heterodimerizing with CXCR4; and (c) acting as a “scavenger” of CXCL12, thus lowering the level of CXCL12 to regulate the activity of CXCR4 [28, 29].

In regard to CXCL12/CXCR7 axis trafficking, it is generally accepted that CXCR7 functions as a scavenger receptor that depletes CXCL12 from the extracellular space, traffics CXCL12 to lysosomes and degrades it, and that recycles CXCR7 back to the cell membrane [30]. This phenomenon has been well elucidated by our group in a human breast cancer cell line MCF-7, which is known as endogenously expressed CXCR7 on the cell membrane (data not shown) [31]. However, the recycling machinery could only cope with endogenously expressed, but not with forcedly, overexpressed CXCR7 [5]. This may explain why our constructed overexpressed CXCR7 A549-GFPLuc-CXCR7 lung cancer cells were treated with certain concentration of CXCL12, with CXCR7 recycling not observed. In addition, overexpression of CXCR7 increases expression of CXCR4 on the cell surface compared with non-transduced A549 lung cancer cells (data not shown). This observation is coincident with previous studies that CXCR7 can maintain the cell surface levels of CXCR4 in the presence of constant concentration of CXCL12, preventing the internalization of CXCR4 in breast cancer cells. This founding may be interpreted as that CXCR4 and CXCR7 can often each form homodimers and/or heterodimers depending on the expression level of both receptors, suggesting that CXCR7 could be a requirement for potentiating CXCR4/CXCL12 signaling [32]. The balance of CXCR7 and CXCR4 has been described during liver regeneration and fibrosis. In response to injury, CXCR7 pathway in liver sinusoidal endothelial cells (LSECs) plays an indispensable role in stimulating regeneration. After repeated stimulation, CXCR7 pathway in LSECs was counterbalanced by CXCR4 upregulation, which would lead to fibrosis [33].

In addition, many studies have suggested that CXCR7 promotes cell proliferation, adhesion, migration, survival, angiogenesis and cell metastasis in various cancers [34, 35]. In the present study, our data have documented that CXCR7 enhances cell migration, invasion and polarization in vitro, and promotes primary lung tumor growth and metastases in vivo. In terms of the relationship between polarization and migration, cell polarization initiates cell migration. Rac1 is a member of the Rho family small signaling G protein and regulates cytoskeleton reorganization. In polarization, Rac1 activation initiates cell mobility through the formation of the lamellipodial protrusion for migration, see Fig. 5. It was found that CXCL12 binding increased phosphorylated AKT (Ser473) in endothelial cells, suggesting involvement of the PI3K pathway, and that CXCL12 also promoted the activation of Rac1 [36]. Moreover, Y-C Wu et al. reported that high TGFβ1 and CXCR7 expression was strongly connected with the poor survival rate in lung adenocarcinoma patients. CXCR7 is up-regulated most compared with other chemokine receptor by TGFβ1 in human lung adenocarcinoma A549 cell line. CXCR7 silencing significantly represses cancer cell migration, invasion and epithelial-mesenchymal transition (EMT) induced by TGFβ1 in vitro, as well as sphere-forming capacity, stem-like properties, chemoresistance and TGFβ1-induced CSC tumor initiation in vivo [26]. Recently, it has been found that when KRAS-driven non-small cell lung cancer (NSCLC) patients were treated with epidermal growth factor receptor (EGFR) targeting therapeutic approach, significantly higher expression of CXCR7 were observed and MAPK (ERK1/2) signaling was activated, implying that EGFR and CXCR7 had a crucial interaction in NSCLC [37]. Furthermore, Becker JH et al. considered that CXCR7 inhibition could prevent the emergence of acquired EGFR tyrosine kinase inhibitor (TKI) resistance in EGFR mutant NSCLC with an EMT phenotype [38]. In this study, we systematically investigate the role of CXCR7 independent of CXCR4 in lung tumor growth and metastasis. Based on the fact that the expression of CXCR7 and CXCL12 was significantly upregulated in metastatic organs than in primary tumors, we speculate that the mechanism of CXCR7 enhancing lung cancer metastasis may include following steps. Firstly, primary lung cancer cells can secrete exosomes which could induce pre-metastatic niche formation and increase the expression of CXCL12. Then secreted CXCL12 enters into the blood circulation and directs CXCR4/CXCR7+ lung cancer cells to the sites of targeted organs. Finally, the metastatic lung cancer cells proliferate in the colonized organs to form metastatic sites [39]. Thus it can be concluded that CXCR7 expression level shows striking differences during pathological processes especially in malignancies, which might relate to their augmented growth and migration capacities and in line with an advantage of metastasis.

## Conclusion

Our results suggested that CXCR7 facilitated cell migration, invasion and polarization in A549 lung cancer cell line, and promoted lung cancer cell derived tumor growth and metastasis in SCID/Beige mice. These findings advance our understanding about the function of CXCR7 in multiple behavioral phenomenon of lung cancer. Despite this, it seems that how CXCR7 evokes these effects and interacts with its downstream signal transduction proteins differs depending on the disparate cancer cell types investigated. Nevertheless, questions still remain about the mechanisms by which CXCR7 mediates enhanced cell migration, invasion and polarization, as well as proliferative and metastatic advantages in lung cancer. Further experiments and studies by our group and others are ongoing to elucidate the specific pathways and mechanisms of CXCR7 mediating its effects in lung cancer. However, it is worth pointing out that this knowledge would guide ongoing development and suggest the likelihood of therapeutic benefit for selectively targeting CXCR7 rather than CXCR4 to treat lung cancer.

## Data Availability

The datasets generated and analysed during the current study are not publicly available because that it also forms part of another ongoing study, but are available from the corresponding author on reasonable request.

## Abbreviations

CXCL12: Chemokine (C-X-C motif) ligand 12
SDF-1: Stromal cell-derived factor-1
CXCR4: C-X-C chemokine receptor type 4
CXCR7: C-X-C chemokine receptor type 7
LDCT: Low dose helical computed tomography
TCSCs: Tissue-committed stem cells
GPCR: G-protein coupled receptor
NSCLC: Non-small-cell lung cancer
MLN: Metastatic lymph nodes
DFS: Disease-free survival rate
HUVEC: Human umbilical vein endothelial cells
EMT: Epithelial-mesenchymal transition
EGFR: Epidermal growth factor receptor
TKI: Tyrosine kinase inhibitor
